# Proteomic characterization of secretory granules in dopaminergic neurons indicates chromogranin/secretogranin-mediated protein processing impairment in Parkinson’s disease

**DOI:** 10.18632/aging.203415

**Published:** 2021-08-21

**Authors:** Gehua Wen, Hao Pang, Xu Wu, Enzhu Jiang, Xique Zhang, Xiaoni Zhan

**Affiliations:** 1 School of Forensic Medicine, China Medical University, Shenyang, PR China; 2 Department of Geriatrics, The First Affiliated Hospital of China Medical University, Shenyang, PR China

**Keywords:** Parkinson's disease, secretory granules, secretogranin, proteomics, lipid metabolism, neuropeptide

## Abstract

Parkinson’s disease (PD) is an aging disorder related to vesicle transport dysfunctions and neurotransmitter secretion. Secretory granules (SGs) are large dense-core vesicles for the biosynthesis of neuropeptides and hormones. At present, the involvement of SGs impairment in PD remains unclear. In the current study, we found that the number of SGs in tyrosine hydroxylase-positive neurons and the marker proteins secretogranin III (Scg3) significantly decreased in the substantia nigra and striatum regions of 1-methyl-4-phenyl-1, 2, 3, 6-tetrahydropyridine (MPTP) exposed mice. Proteomic study of SGs purified from the dopaminergic SH-sy5Y cells under 1-methyl-4-phenylpyridinium (MPP+) treatments (ProteomeXchange PXD023937) identified 536 significantly differentially expressed proteins. The result indicated that disabled lysosome and peroxisome, lipid and energy metabolism disorders are three characteristic features. Protein-protein interaction analysis of 56 secretory proteins and 140 secreted proteins suggested that the peptide processing mediated by chromogranin/secretogranin in SGs was remarkably compromised, accompanied by decreased candidate proteins and peptides neurosecretory protein (VGF), neuropeptide Y, apolipoprotein E, and an increased level of proenkephalin. The current study provided an extensive proteinogram of SGs in PD. It is helpful to understand the molecular mechanisms in the disease.

## INTRODUCTION

Parkinson’s disease (PD) is the second most common age-related neurodegenerative disorder. The classical pathological characteristics of PD are progressive injury and loss of dopaminergic neurons in the substantia nigra pars compacta (SNpc) and the formation of Lewy bodies and Lewy neurites [1, 2]. The molecular mechanisms underlying PD are yet poorly understood due to a large number of factors involved and their complicated interplay [3–8]. Merging studies implicate dysfunction of vesicle transport as an underlying mechanism of PD. The abnormal fusion and cycling of synaptic vesicles (SVs) are found at presynaptic terminals of the dopaminergic neuron [9, 10]. Without proper maintenance of SV trafficking, endocytosis, and macroautophagy, synapses become dysfunctional, leading to impaired secretion of dopamine and neurodegeneration [2, 9].

Insights from electron microscopy studies show there are a large number of dense-core vesicles (DCVs) besides SVs in the neurons and the neuroendocrine cells, which are so-called secretory granules (SGs) or secretory vesicles [11, 12]. Recently, abundant SGs have been identified in the thalamocortical axons, hippocampal glutamatergic neurons, and striatal GABAergic neurons *in vitro* [13]. The nervous system utilizes SGs for the regulated secretion of neuropeptides and neurotrophins, for instance, catecholamines, somatostatin, neuropeptide Y (NPY), and brain-derived neurotrophic factor (BDNF). These neurotransmitters play essential roles in cognitive, emotion, neurogenesis, and cell-cell communication [11, 14]. SGs represent the primary subcellular site for the biosynthesis, storage, and release of neuropeptides and hormones. They are filled with cargo at the trans-Golgi network to form primary vesicles, undergo maturation step as they travel along cytoskeletal filaments, and finally release the processed proteins responding to calcium signals. SGs secretory and processing machinery require orchestrated actions of an incredible repertoire of lipids, ATP, cytoskeletal filaments, and enzymes [12]. Currently, alterations in SGs in the brain are associated with many neurological and psychiatric disorders [15, 16]. However, whether there is a high density of SGs in the dopaminergic neuron and their involvement in the pathogenesis and progression of PD has yet to be determined.

Secretogranins II (Scg2) and Secretogranins III (Scg3) are members of the secretogranin family, a class of acidic secretory proteins abundantly expressed on SGs. They are traditionally considered markers for SGs and the regulated secretory pathway and act as critical molecules in the early aggregation and processing of peptide hormones in the vesicles. Scg2 participates in the formation of SGs-like structures and packaging of neuropeptides into SGs. Its expression is required to maintain the normal morphology and number of SGs [17, 18]. Scg3 acts as a bridge between chromogranin A (CgA) and the cargo aggregates. It also participates in early peptide processing by interacting with carboxypeptidase E (CPE) in the vesicle membrane [19, 20]. In our previous studies, a toxin-induced by dopaminergic neuron model of PD was established with paraquat (PQ). We observed that the number of Scg3 positive SGs in the dopaminergic SH-sy5y cells was significantly reduced. And the residual vesicles aberrantly accumulated in the perinuclear endoplasmic reticulum (ER) [21]. Also, Scg3-positive SGs were involved in the transport and metabolism of parkinsonian toxin PQ, leading to the injury of multiple intracellular organelles in dopamine neurons [22]. Based on the above results, we suppose the dysfunctional transport of SGs and secretion of cargos they carry are possibly involved in the development of PD. Therefore, in the current study, SGs in the dopaminergic neurons were examined *in vivo* on the MPTP-induced mouse model of PD. And the liquid chromatography-tandem mass spectrometry (LC-MS/MS) and proteome analysis were adopted to illustrate the molecular mechanisms of SGs disorders in the pathology of PD.

## MATERIALS AND METHODS

### Antibodies and reagents

MPTP hydrochloride (MPTP · HCl) (M0896) and MPP^+^ iodide (MPP^+^· I) (D048) were obtained from Sigma Chemical Company (St. Louis, MO). Dulbecco’s modified Eagle’s medium/F12 (DMEM/F12), penicillin, streptomycin, and fetal bovine serum (FBS) were from Gibco (Grand Island, NY). Mouse anti-EEA1 (ab70521) and mouse anti-mitochondrial (MTC02) (ab3298) antibodies were purchased from Abcam (New Territories, Hong Kong). Mouse anti-calnexin (sc-46669), rabbit anti-Sg II (sc-50290), and Sg III (sc-50289) antibodies were purchased from Santa Cruz Biotechnology (Santa Cruz, CA, USA). Mouse anti-golgin-97 (human)-M (A21270), Alexa Fluor 488 donkey anti-mouse IgG, and Alexa Fluor 594 donkey anti-rabbit IgG (ab150076)-conjugated secondary antibodies were purchased from Thermo Fisher Scientific (Waltham, MA, USA). The MTS CellTiter 96^®^Aqueous One Solution Reagent was from Promega (Madison, WI, USA). RNAiso Plus and SYBR Prime EX Taq were from TaKaRa Biotechnology (Shiga, Japan). Evo M-MLV RT Premix was from Accurate Biotechnology (Hunan, China). The BCA Protein Assay Kit and Easysee reagent were from Beyotime Biotechnology (Beijing, China) and TransGen Biotech (Beijing, China).

### Animals and drug administration

Male C57BL/6 mice (8 weeks, 22–25 g) were from the Department of Laboratory Animal Science of China Medical University. All mice were raised in an environment on a 12-h light/dark cycle with free access to water and food. All experiments were conducted according to the National Institutes of Health guide for the care, and use of laboratory animals (NIH Publications No. 8023, revised 1978) and were approved by the Animal Care Committee of the China Medical University. Mice adapted for one week and were pre-trained for the behavioral tests. Mice from MPTP and control groups were injected with MPTP hydrochloride (Sigma-Aldrich, St Louis, MO, USA, dissolved in 0.9% saline, 30 mg/kg, i.p.) and an equal volume of normal saline for five consecutive days, respectively. Animals were sacrificed on the 7th day after the last injection for further study.

### Immunohistochemistry

The sections of SNpc and corpus striatum (CPU) were processed for immunohistochemistry of tyrosine hydroxylase (4 sections per mouse). Paraffin sections were initially de-waxed in xylene and dehydrated through graded alcohols. For antigen retrieval, sections were incubated in 0.01 M citrate buffer (pH 6.0) by microwaving and the endogenous peroxidase was blocked by 3% hydrogen peroxide (H_2_O_2_) for 30 minutes. All samples were blocked with 5% goat serum in PBS to avoid nonspecific antibody binding before incubation with the primary antibody at 4°C overnight. After washing in PBS, these sections were subsequently processed with the biotinylated goat anti-rabbit secondary antibody for 30 minutes. After that, the slides were incubated with horseradish peroxidase (HRP)-labeled streptomycin for 30 minutes at room temperature. The Avidin–peroxidase protocol was applied for visualization using diaminobenzidine (DAB) as chromogen and H_2_O_2_ as substrate. For immunofluorescence analysis, tissue sections were first incubated with 10% donkey serum blocking solution for 1 h and then exposed to the primary anti-Scg2 (1:250) and Scg3 antibody (1:200) diluted in 5% blocking solution overnight. Tissue sections were subsequently incubated with donkey anti-mouse AlexaFluor488 (1:400). And donkey anti-rabbit AlexaFluor594 antibody (1:400) for 2 h at room temperature after washing with PBS. After washing three times with PBS, tissue sections were mounted using the Antifade Mounting Medium (Beyotime, Shanghai, China). Images were acquired using an Axio Scan.Z1 automatic digital glass slide scanning system (Zeiss, Jena, Germany). The mean of immunoreactivity was analyzed in areas of interest (ROI) in five four sections per mouse and quantified as the integrated density using the ImageJ software 6.0 (National Institutes of Health).

### Cell culture and treatment

Human neuroblastoma SH-sy5Y cells were maintained in Dulbecco’s modified Eagle’s medium (DMEM)/F12 (Gibco, Grand Island, NY, USA) supplemented with 10% fetal bovine serum (Hyclone, Logan, UT, USA), 100 U/mL penicillin, and 100 μg/mL streptomycin in a 5% CO_2_ atmosphere at 37°C. Cells were seeded in 10 cm^2^ culture dishes at a concentration of 5×10^4^ cells/cm^2^ and treated with either fresh medium alone or medium containing various concentrations of MPP^+^ (0-2mM) 24 h later.

### MTS assay

Cell viability was measured by CellTiter 96^®^ Aqueous One Solution Reagent (MTS, Promega, Madison, WI, USA) as described by our previous study. Briefly, approximately 1 × 10^4^ cells/well were seeded in a 96 well-plate, and 120 ul mixture (20 ul MTS reagent and 100 ul medium) was added after the treatment is completed. After incubated for 1 h at 37°C, the absorbance at 490 nm was measured in a SpectraMax M2 plate reader (Molecular Devices, San Jose, CA, USA).

### Subcellular fractionation for secretory granules purification

The subcellular fractionation for SGs in SH-sy5Y cells was isolated using a sucrose gradient according to the method in the previous study [20]. In detail, cells were harvested in phosphate-buffered saline (PBS, containing 10 mM EDTA) at 37°C and passed through a 27-gauge needle 6 times. The pellets were then homogenized in 250 mM sucrose (containing 4 mM HEPES (pH 7.4), 1 mM MgCl_2_, 0.005% DNase, and a protease inhibitor mixture (PPC1010, Sigma-Aldrich, Darmstadt, Germany) with a homogenizer. The homogenate was centrifuged at 3,000 g for 2 min. The resulting supernatant was centrifuged at 5,000 g for 15 min at 4°C. The postnuclear supernatant was recentrifuged at 26,000 g for 15 min and resuspended in the homogenization buffer. The crude organelle fraction was then layered to a sucrose density gradient (20%-70%) and centrifuged at 113,000 g 18 h at 4°C using an SW32 swing rotor (Beckman Coulter, Pasadena, CA, USA). Gradients were fractionated by piston displacement from top to bottom. The experiment was conducted independently three times.

### Transmission electron microscopy

The substantia nigra (SN) was cut into 1 mm^3^ blocks and fixed in 2.5% glutaraldehyde fixative and 1% osmium tetroxide fixative for 2 hours. Specimens were dehydrated, soaked, embedded, and then cut into slices of 1–10 μm thickness using an ultra-thin slicer. For the observation of SGs that were purified via sucrose density gradient centrifugation, the 1.5 mL of sucrose layer was washed with PBS and centrifuged at 113,000 g 3 h at 4°C to collect the pellets. Pellets were resuspended in 4% paraformaldehyde in PBS. Sub-sequently, a formvar-carbon-coated grid was floated on 15 μL sample droplets on a parafilm for 10 min. And then the grid was floated on 15 μL water for 5 min. The grid was floated on 20 μL of 2% uranyl acetate in water for 30 s for negative stain. The excess fluid from the grid was eliminated with a filter paper and the grid was dried on filter paper for another 5 min. Ultimately, the samples were viewed on a transmission electron microscope (Hitachi H-7650) at 80 kV, and micrographs were taken at 80, 000× magnification.

### Western blot analysis

Cells from treated and control groups were incubated in an FBS-free medium either contains MPP^+^ or not for 24 h. Cells were lysed in RIPA buffer with PMSF and quantified using a BCA assay kit (Beyotime, China). Meanwhile, the medium containing secreted proteins of cells was collected as the conditioned medium (CM) after removing dislodged cells by centrifugation. A total of 20 ug cell lysates or 10 uL conditional medium was loaded for each line and quantified by densitometric analysis normalized to β-actin and total protein in CM measured by the Coomassie blue staining, respectively. For the subcellular fractionations, 400 uL of the fractionated samples were precipitated in the methanol-chloroform method for further SDS-PAGE analysis. Standard western blotting techniques were conducted as in our previous study. Total protein was separated via 10% SDS-PAGE and transferred to polyvinylidene difluoride (PVDF) membranes. Then the membranes were blocked with 5% nonfat milk for 2 h at room temperature and incubated with primary antibodies at 4°C overnight. Primary antibodies used were rabbit anti-Scg3, rabbit anti-Scg2, mouse anti-EEA1, mouse anti-Mito, mouse anti-Calnexin, mouse anti-calnexin, and rabbit anti-β actin. Proteins were detected using infrared emitting fluorophore dyes coupled to the horseradish peroxidase (HRP) secondary antibodies.

### Liquid chromatography-tandem mass spectrometry (LCMS/MS) analysis

The indicated sucrose gradient (8th layer from the top), which contains the highest density of SGs was collected for label-free quantification proteomics analysis. Briefly, the subcellular fractionations were washed with PBS three times, and protein (200 μg each) from pellets was extracted using UA buffer (8 M Urea, 150 mM Tris-HCl pH8.0) and quantified with a BCA Assay Kit (BioBio-Rad, Hercules, CA, USA). The LC-MS/MS was performed on a Q Exactive Plus mass spectrometer and the Easy 1200 nLC liquid chromatography system (Thermo Fisher Scientific, Waltham, MA, USA) after a filter-aided sample preparation (FASP) method. The mass spectrometry proteomics data have been deposited to the ProteomeXchange Consortium via the PRIDE partner repository with the dataset identifier PXD023937 (reviewer account username: reviewer_pxd023937@ebi.ac.uk, password: hmf8J2qB) [23]. The data were further analyzed with MaxQuant software (version 1.6.0.16) and searched against the UniProt database for Homo sapiens (Human) (192,367 total entries, downloaded 01/08/2020). Proteins with a fold change of ≥ 1.5 and a *P*-value of < 0.05 were considered significantly differentially expressed (DEPs). Analyses of the bioinformatics data were carried out with Perseus and *R* statistical computing software. For sequence annotation, information was extracted from the UniProtKB/Swiss-Prot database (https://www.uniprot.org/), the Kyoto Encyclopedia of Genes and Genomes (KEGG) (https://www.kegg.jp/), and the Gene Ontology (GO) resource (http://geneontology.org/). Construction of protein-protein interaction (PPI) networks was also conducted by using the online STRING database (https://www.string-db.org/) with high confidence (0.700) as the threshold.

### Quantitative polymerase chain reaction (qPCR) validation

Total RNA was isolated and reverse transcribed to cDNA with the Evo M-MLV RT Premix according to the manufacturer’s instructions. The expressions of the significantly differentially expressed proteins in the secretory vesicles were validated by quantitative polymerase chain reaction (qPCR). The cDNA was amplified using an SYBR Prime EX Taq Kit with the primers listed in Supplementary Table 1. The qPCR was performed using an ABI 7500 Real-Time PCR System, and data analysis was performed using the ΔΔCt method. Triplicate plates were tested for each sample, and the experiment was repeated three times.

### Statistical analysis

For statistical analysis, the data were expressed as means ± standard deviation (SD). All analyses were carried out using the GraphPad Prism 7.0 Software (San Diego, CA, USA), and two-tailed unpaired Student *t*-tests were performed between control and treated groups. *P* values <0.05 were considered significant.

## RESULTS

### Scg3 was reduced in the substantia nigra and striatum in the mouse model of MPTP-induced Parkinson’s disease

**Figure 1:**
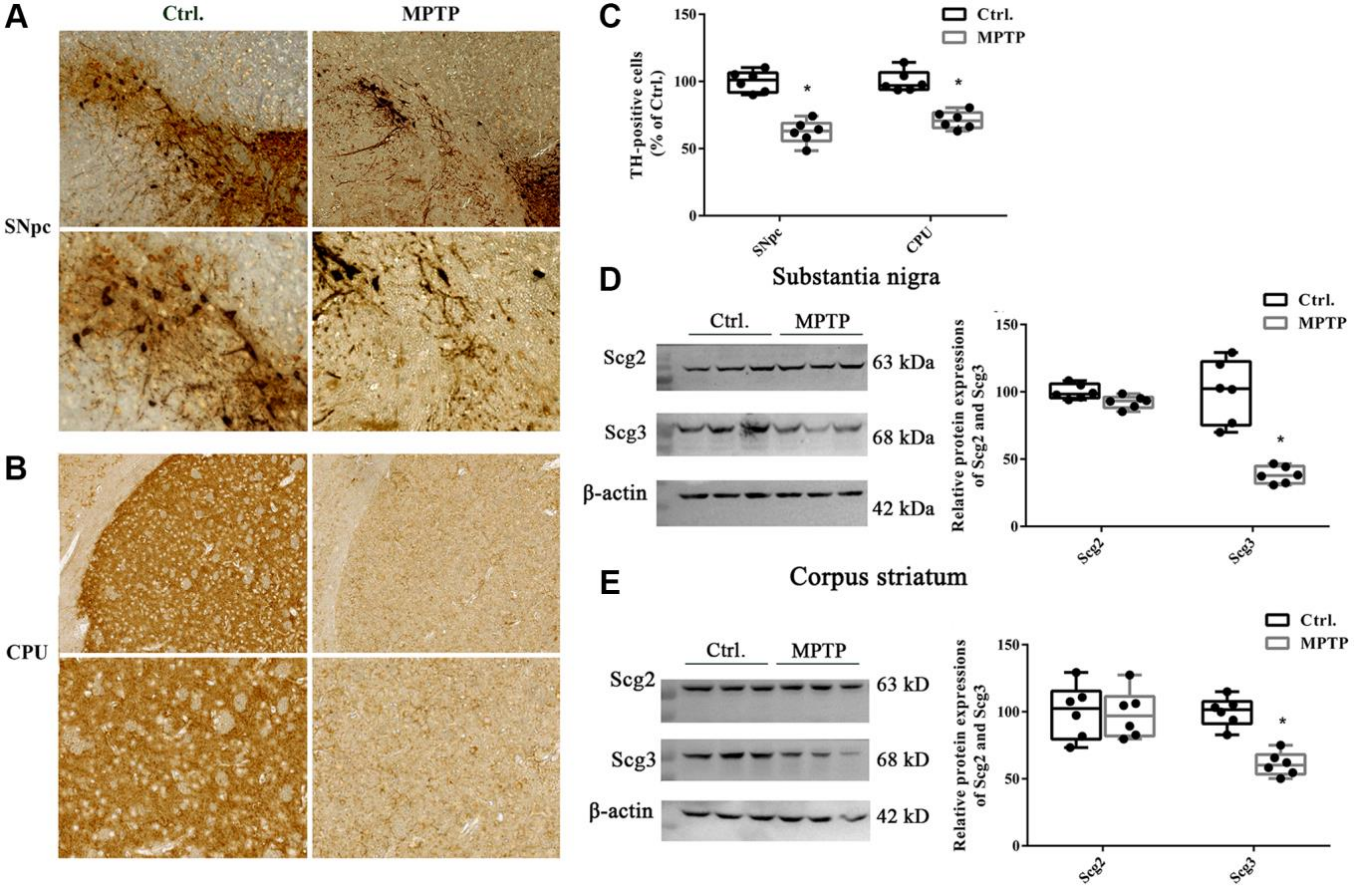
**Immunohistochemistry of tyrosine hydroxylase positive neurons and levels of secretogranins in the substantia nigra and corpus striatum.** (**A**–**B**) Tissues were immunostained for tyrosine hydroxylase (TH) in both cell bodies in substantia nigra pars compacta (SNpc) and fibers and terminals in the corpus striatum (CPU) in MPTP-treated and control groups. (**C**) (TH)–positive stains in MPTP-treated and control groups were assessed by mean optical density via Image J software. (**D**–**E**) Scg2 and Scg3 protein expressions in SN and CPU were analyzed by immunoblotting and quantified by densitometric analysis normalized to GAPDH. Two-tailed unpaired Student *t*-tests were performed between the control and treated groups. ^*^Statistically significant with *P* < 0.05; Error bars are SD; *N* = 6.

To evaluate whether the MPTP-induced PD model was successfully established, the number of dopaminergic neurons in the SN and CPU were assessed by the immunohistochemistry of tyrosine hydroxylase (TH). The results showed that MPTP lesion for 5 successive days significantly decreased TH-positive cell bodies in SN and TH-positive cell terminals in the CPU compared with the control group (62.2% and 71.17% of control, respectively), suggesting there were an injury and loss of dopaminergic neurons after MPTP exposure (Figure 1A–1C). Meanwhile, a significantly decreased level of Scg3, which was considered as marker proteins of the SGs, was observed in both SN and CPU regions of the PD mouse model. In contrast, the level of Scg2 remained unchanged. In addition, we found that the reduction of Scg3 in SN was more than that in the CPU. (Figure 1D–1E). Taken together, the above results show that the expressions of Scg3 decreased in the SN and CPU in the MPTP-induced PD model, suggesting the SGs may be impaired in the injury dopamine (DA) neurons.

### Secretory granules were decreased in the injured nigrostriatal dopaminergic neurons in the mouse model of Parkinson’s disease

**Figure 2:**
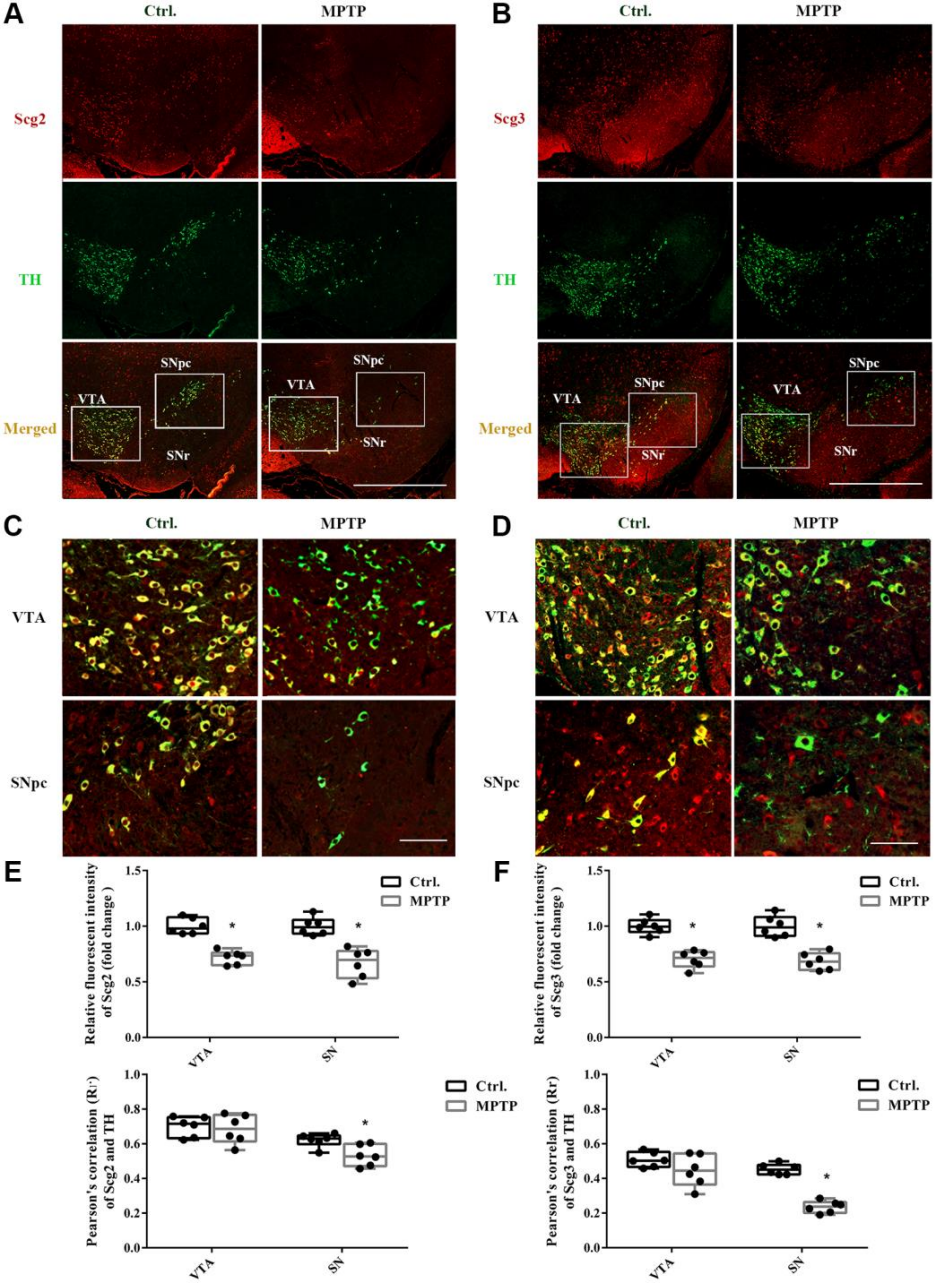
**Double immunofluorescence staining of Scg2 or Scg3 with TH-positive DA neurons.** (**A**–**B**) Co-staining of Scg2 or Scg3-positive secretory granules (Red) with TH-positive DA neurons (Green) at low magnification (20×), Scale bar, 50 mm. (**C**–**D**) Co-staining of Scg2 or Scg3-positive secretory granules (Red) with TH-positive DA neurons (Green) in the ventral tegmental area (VTA) and substantia nigra par compacta (SNpc) at high magnification (40×), Scale bar, 50 mm. (**E**–**F**) Scg2 or Scg3 protein expressions in substantia nigra of control and MPTP-induced PD mice were quantified by mean fluorescence. And the quantification of colocalization between Scg2 or Scg3 and TH was determined by Pearson’s correlation coefficient using the Image Pro Plus software. Two-tailed unpaired Student *t*-tests were performed between the control and treated groups. ^*^Statistically significant with *P* < 0.05; Error bars are SD; *N* = 6.

Abundant SGs have been identified in various endocrine and neuroendocrine cells and tissues in previous studies, for instance, chromaffin cells, insulin beta-cells, pituitary, and adrenal glands [24, 25]. However, the SGs in the DA neurons have rarely been studied. To determine whether SGs were affected in the injury DA neurons, double immunostaining was carried out for the secretogranins and TH in SN. As shown in Figure 2, both Scg2 and Scg3 were highly expressed in SN by and large, including SNpc and substantia nigra pars reticulate (SNr). Moreover, Scg3 seemed to show a more comprehensive distribution compared to Scg2. In the TH-positive DA neurons in the ventral tegmental area (VTA) and SNpc, we observed plenty of red particles labeled by the antibodies of two secretogranins in the cell bodies (Figure 2A–2D). The existence of SGs was also confirmed by the electron microscopy of neurons of SN. It showed that several dense-core SGs are present at the border of synaptic vesicle (SV) clusters (Supplementary Figure 1), which have a similar appearance to that found in the hippocampus neurons in the previous study [13]. The overlapping between two secretogranins and TH were further measured by Pearson’s correlation coefficient analysis, in which 0.0–0.2 and 0.2–0.4 are respectively considered as no and weak correlations, whereas 0.4–0.6 and 0.6–0.8 are determined as moderate and strong correlations. According to the results, there was a strong correlation between Scg2 and TH (VTA: 0.70; SNpc: 0.62) and a moderate correlation between Scg3 and TH (VTA: 0.50; SNpc: 0.45). Furthermore, the colocalization index in VTA was generally higher than that of SNpc. However, after insulted by MPTP, the expressions of both Scg2 and Scg3 were significantly decreased in the VTA and SNpc DA neurons according to their fluorescence intensities. Furthermore, a more remarkable decline of correlation between both secretogranins and TH was found in the injured SNpc DA neurons than VTA. The decrease of Scg3 was more notable than Scg2 (Figure 2E–2F). These results were mostly consistent with the immunoblotting tests. They gave further evidence that the secretory granules were decreased in the injured DA neurons in the mouse model of PD, especially DA neurons in SNpc. Also, it suggests the secretory granules might be more vulnerable and susceptible to damage in SNpc DA neurons.

### Expressions and secretions of secretogranins were dysregulated in SH-sy5Y cells under acute and mild MPP^+^ treatment

**Figure 3:**
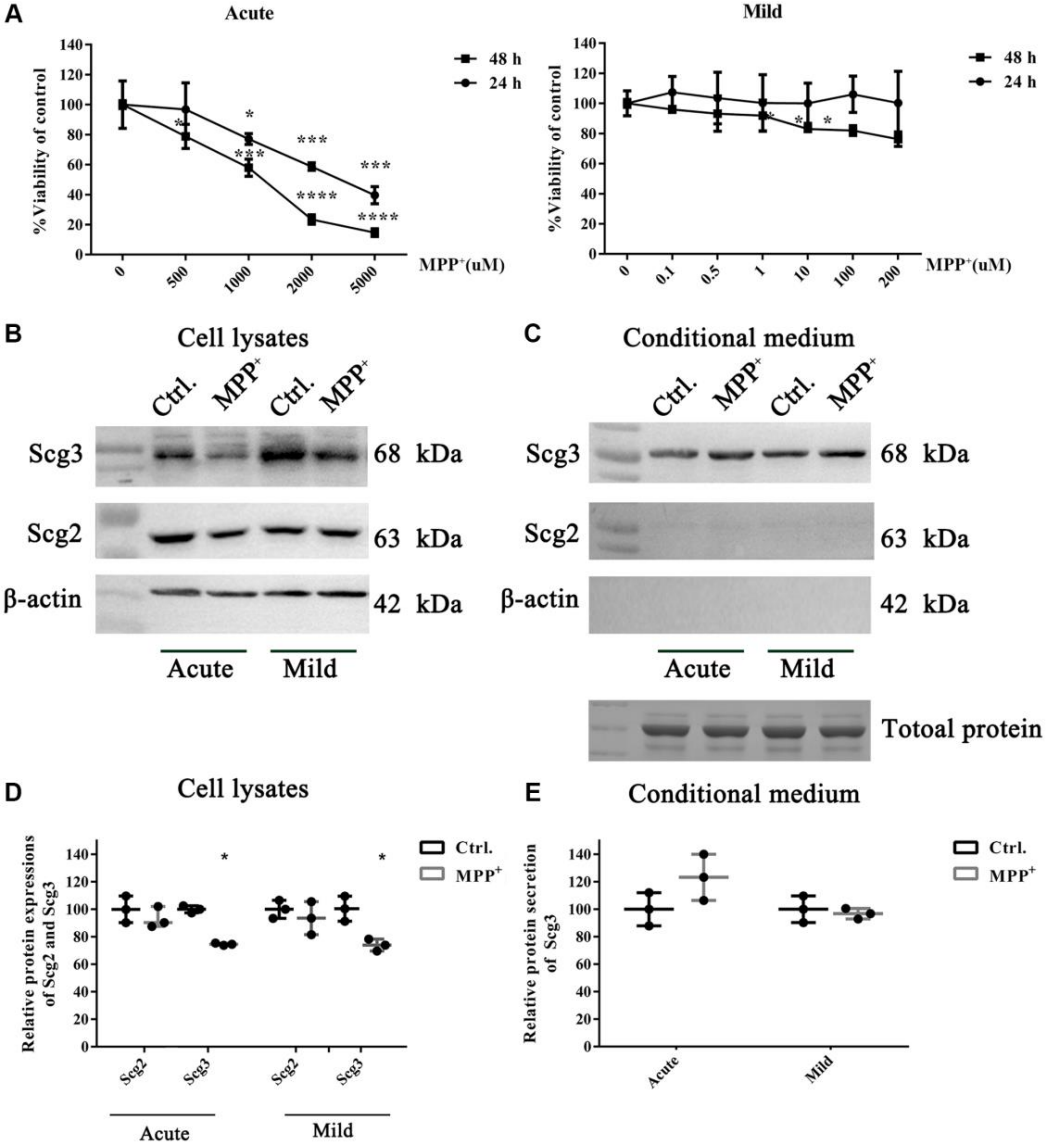
**The effects of acute and mild MPP+ treatments on the cell viability and expressions of secretogranins in SH-Sy5Y cells.** (**A**) SH-Sy5Y cells were exposed to an acute (500–5000 μM) and a mild (0.1–200 μM) treatment of MPP^+^ for 24 and 48 h. The cell viability was measured using the MTT assay. And appropriate treatments were determined as 1000 μM for 24 h and 10 μM for 48 h, respectively. Data from three individual experiments were expressed as the mean percentage of the controls. (**B**) Intracellular protein expressions of Scg2 and Scg3 in the dopaminergic SH-sy5Y cells under acute and mild MPP+ treatments were analyzed by immunoblotting and quantified by densitometric analysis normalized to β-actin. (**C**) The secretions of Scg2 and Scg3 in the conditioned medium of SH-sy5Y cells were analyzed by immunoblotting. According to the Coomassie staining, an equal amount of total protein in the conditioned medium was loaded for each sample. (**D**) The quantifications of intracellular Scg2 and Scg3 levels in the dopaminergic SH-sy5Y cells under acute and mild MPP^+^ treatments. (**E**) The quantifications of Scg3 secretion levels in the dopaminergic SH-sy5Y cells under acute and mild MPP^+^ treatments. Two-tailed unpaired Student *t*-tests were performed between the control and treated groups. ^*^Statistically significant with *P* < 0.05; Error bars are SD; *N* = 3.

To understand the molecular mechanism of SGs impairment in PD. We adopted two MPP^+^-induced cell models that were reported in the previous studies [26]. Dopaminergic SH-sy5Y cells were respectively exposed to high (500–5000 μM) and low (0.1–200 μM) doses of MPP^+^ to establish the acute and mild models, and the cell viability was evaluated by the MTT assay. We found that high dose MPP^+^ exposure caused a concentration-dependent decrease of cell viability, and the acute treatment for 48 h led to severe damage to the cell. In contrast, low dose MPP^+^ exposure for 24 h showed little impact on the cell viability (Figure 3A). To avoid the influence of cell death in the current study, 1000 μM for 24 h and 10 μM for 48 h, which were the lowest doses capable of inducing a significant decrease of viability in the acute and mild treatments, were finally determined as preferable treatments. The protein expressions of the secretogranins were subsequently tested on two cell models. The immunoblotting results showed that Scg3 was remarkably decreased, while the level of Scg2 remained unchanged under mild MPP^+^ treatment. Nevertheless, MPP^+^ acute treatment caused a significant and insignificant reduction of Scg3 and Scg2, respectively. In all, the alternation of Scg3 in the cell model was more evident than Scg2 (Figure 3B, 3D), which was consistent with the results observed *in vivo*. Meanwhile, we investigated the secretions of Scg2 and Scg3 in the conditioned medium (CM). Although Scg2 was highly expressed in SH-sy5Y cells, its secretion was barely observed in the CM. In contrast, there was a slight rise of Scg3 levels under the stimulus of both acute and mild MPP^+^ treatments (Figure 3C, 3E). Based on the above results, the cell under acute MPP^+^ exposure was selected to manipulate Scg2 and Scg3-positive SGs’ decrease in the DA neurons *in vivo*. The model was used in the subsequent analysis. In short, MPP^+^ exposure down-regulated secretogranins, particularly Scg3 in SH-sy5Y cells, further suggesting the SGs are injured in DA neurons in cell models of PD.

### Secretory granules from SH-sy5Y cells were relatively enriched via sucrose density gradient centrifugation

**Figure 4:**
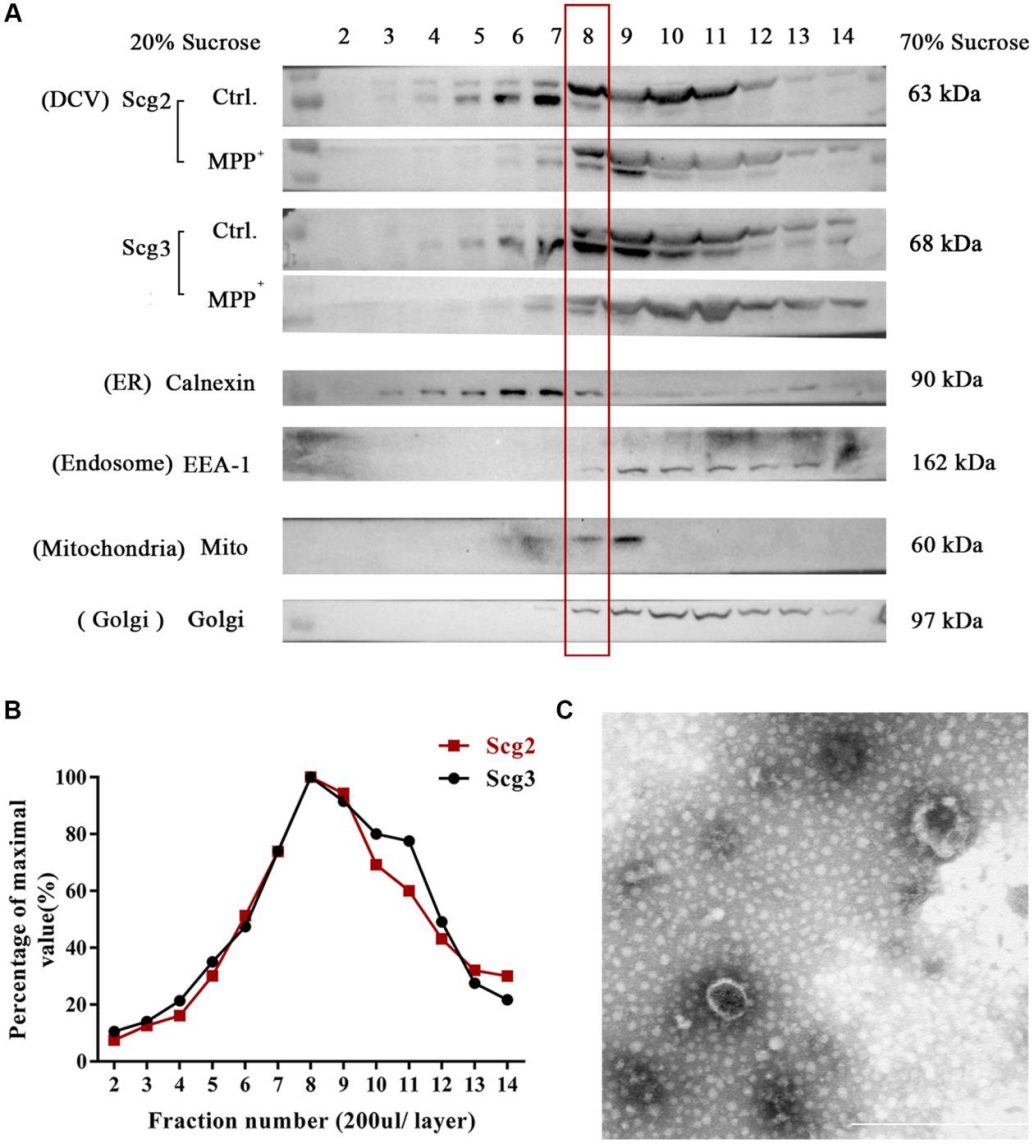
**Fractionation of components in SH-sy5y cells via sucrose density gradient centrifugation.** (**A**) The extract of SH-sy5Y was fractionated into 17 layers via sucrose density gradient centrifugation. And levels of multiple organelles, secretory granules (Scg2 and Scg3), ER (Calnexin), Golgi (Golgi 97), endosome (EEA-1), and mitochondria (Mito) were measured by immunoblotting for each fraction (layer 2-14). (**B**) The protein levels of Scg2 and Scg3 in fractions 2–14 were quantified and showed in a line graph. And layer 8–9 are peak fractions for both proteins. (**C**) Transmission electron microscopy of the purified secretory granules in the 8th fraction of SH-sy5y cells via sucrose density gradient centrifugation. Scale bar = 500 nm.

To enriched SGs in SH-sy5y, the subcellular organelles of cells were separated from the top to the bottom into 17 fractions using sucrose density gradient centrifugation according to the previous studies [20]. The levels of marker proteins of multiple organelles in each fractionated sample were analyzed by immunoblotting. According to the results, most of the tested organelle markers appeared in layers 2–13 of the sucrose gradient. The marker of ER calnexin was mainly identified in fractions 5–7, while Golgi 97 for Golgi apparatus and EEA-1 for endosome were primarily observed in fractions 9–11. An abundant level of Mito, the marker protein mitochondria, was observed in fractions 7–9, among which the 9th layer contains the highest level (Figure 4A). To evaluate the abundance of the secretory granules, we further quantified the levels of marker protein Scg2 and Scg3. It was found that Scg2 and Scg3 were accumulated heavily in fractions 6–11 and showed a buoyant density at the peak fraction 8–9. In addition, there was a pronounced decrease of both proteins in the fraction 8–9 of SH-sy5y cells compared to the control group after MPP^+^ acute exposure. The result is consistent with the decline of secretogranins observed in the total protein in Figure 3. To select the most enriched fraction of SGs, we had conducted preliminary profiling of the proteins in the 8th and 9th layers. We found that some typical neuropeptides (e.g., NPY) and marker proteins (e.g., CgA) were absent in the latter (data not shown). To avoid the interference of various proteins in ER in the 7th layer, the 8th fraction was finally selected for further analysis (Figure 4B). Moreover, the purified SGs were verified by morphology before proteomic testing. Under the transmission electron microscope, integrated SGs in diameter of 100–200 nm can be observed, which were shown as spherical organelles with an electron-dense core surrounded by an electron-transparent confining membrane, as the previous studies describe [24, 25] (Figure 4C). The above results suggest that the SGs from SH-sy5Y cells were relatively enriched via sucrose density gradient centrifugation.

### Lysosome and peroxisome functions and metabolism of energy and lipid metabolism were affected in the MPP^+^-induced cell model of Parkinson’s disease

In mass spectrometry, 4249 proteins were identified in the SGs enriched samples of control and MPP^+^ groups in total. Among these proteins, 536 were significantly differentially expressed (137 increased, 399 decreased) between the control and MPP^+^ treated groups. Fisher’s exact test for the enrichment of GO protein annotations in the set of significantly different proteins revealed a range of protein categories (FDR < 0.05). These significantly differentially expressed proteins between the control and MPP^+^ treated groups were analyzed and visualized by conducting heatmap analysis using complete linkage hierarchical clustering (Supplementary Figure 2). According to the result, cluster 1 represents significantly upregulated proteins in MPP^+^ treated groups. These proteins mainly participate in the biological process of organonitrogen compound metabolism, regulation of primary metabolism, and macromolecule metabolism. Most of them possess the molecular function of protein binding, including identical protein binding, protein dimerization, and enzyme binding (Supplementary Figure 3). Cluster 2 represents significantly downregulated proteins and mainly participates in the transport, cellular macromolecule metabolic, protein metabolic biological process. And these proteins clustered in the molecular function of nucleic acid binding, including anion binding, nucleotide-binding, and nucleoside phosphate binding. Also, the results of KEGG analysis further revealed that in MPP^+^ treated cells, the anaerobic and anaerobic oxidation of carbohydrates relegated pathway were significantly activated, which is the key process for ATP production and energy providing in cells, for instance, glycolysis/ gluconeogenesis, pentose phosphate pathway, pyruvate metabolism and citrate cycle (TCA cycle). In contrast, the pathways that are related to endocytosis, lysosome, and peroxisome were significantly downregulated. Moreover, we found that the fatty acid metabolism and biosynthesis process was also downregulated in MPP^+^ treated cells, accompanied by DNA replication. These lipid-related enzymes consist of phospholipase D3 (0.5; *P* = 0.02), non-specific lipid transfer protein (0.39; *P* = 0.01), enoyl- CoA hydratase (0.42; *P* = 0.007), cholesteryl ester hydrolase (0.49; *P* = 0.04), phosphatidylinositol 5-phosphate 4-kinase (0.28; *P* = 0.04), phosphoinositide phospholipase C (0.75; *P* = 0.03) and etc., Meanwhile, several fatty acid metabolism and biosynthesis involved pathways were profoundly downregulated in the PD cell model, including biosynthesis of unsaturated fatty acids (hsa01040; *P* = 0.006), fatty acid degradation (hsa00071; *P* = 0.002), fatty acid metabolism (hsa01212; *P* = 0.002), and steroid biosynthesis (hsa00100; *P* = 0.008) (Supplementary Figure 4). In addition, we identified two differentially enriched pathways that were previously reported to contribute to PD: The rap1 signaling pathway (*P* = 0.003) and the mTOR signaling pathway (*P* = 0.002). Taken together, in the MPP^+^ -induced cell model of Parkinson’s disease, the anaerobic oxidation process is active, while the lysosome and peroxisome functions, DNA replicon, and lipid metabolism is slowed down. It suggests that the dopaminergic cells are under a compromised energy metabolic and hypoxic condition, and the clearance function and lipid metabolism are affected when exposed to MPP^+^.

### Multiple categories of proteins in secretory granules are dysregulated and protein processing and transporting were affected in the MPP^+^-induced cell model of Parkinson’s disease

**Figure 5:**
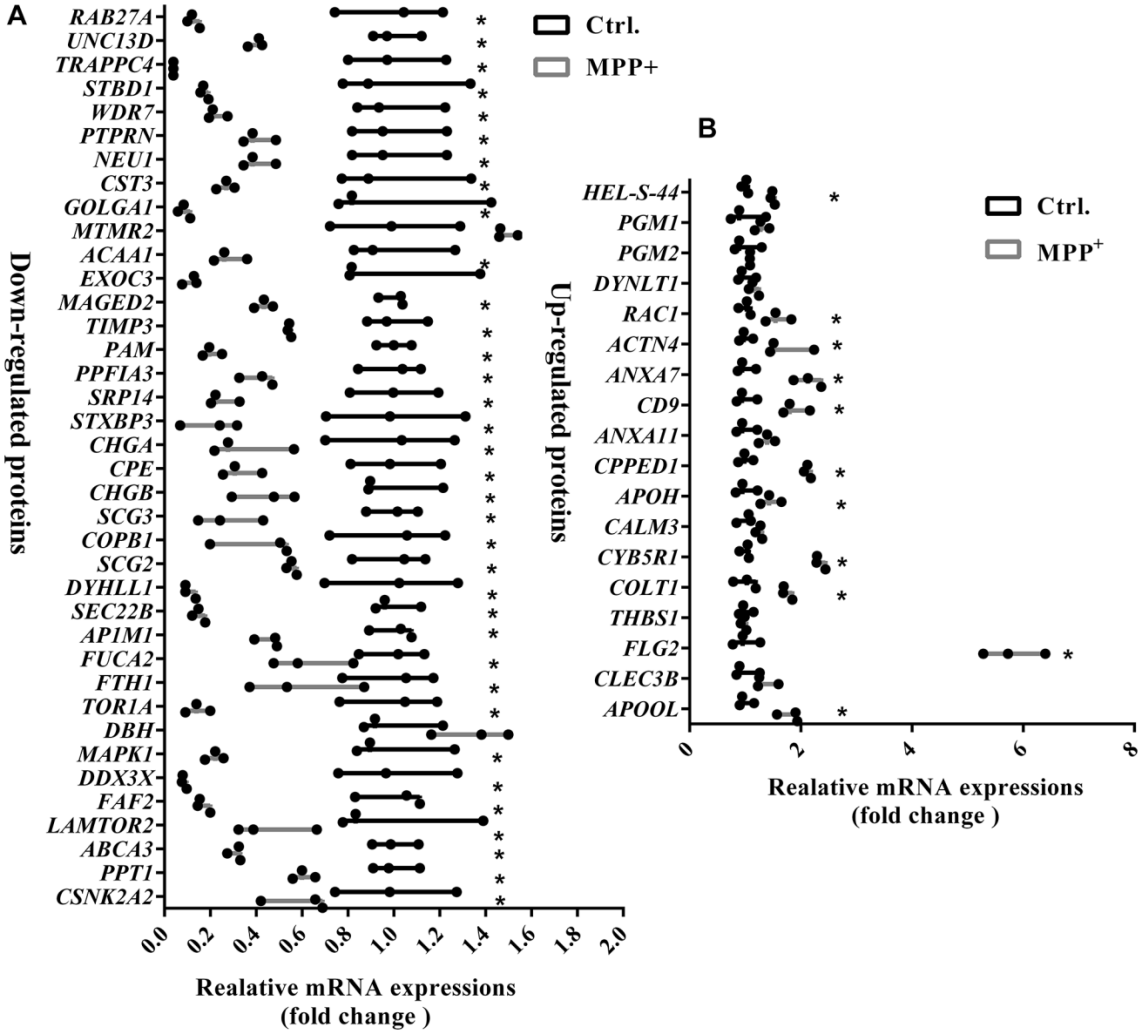
**Verification of mRNA expressions of 56 significantly differentially expressed proteins on the secretory granules between control and MPP+ groups via real-time PCR.** (**A**) The mRNA expressions of 38 significantly down-regulated proteins on the secretory granules were verified using real-time PCR. (**B**) The mRNA expressions of 18 significantly upregulated proteins on the secretory granules were verified using real-time PCR. Two-tailed unpaired Student *t*-tests were performed between the control and treated groups. ^*^Statistically significant with *P* < 0.05; Error bars are SD; *N* = 3.

**Figure 6:**
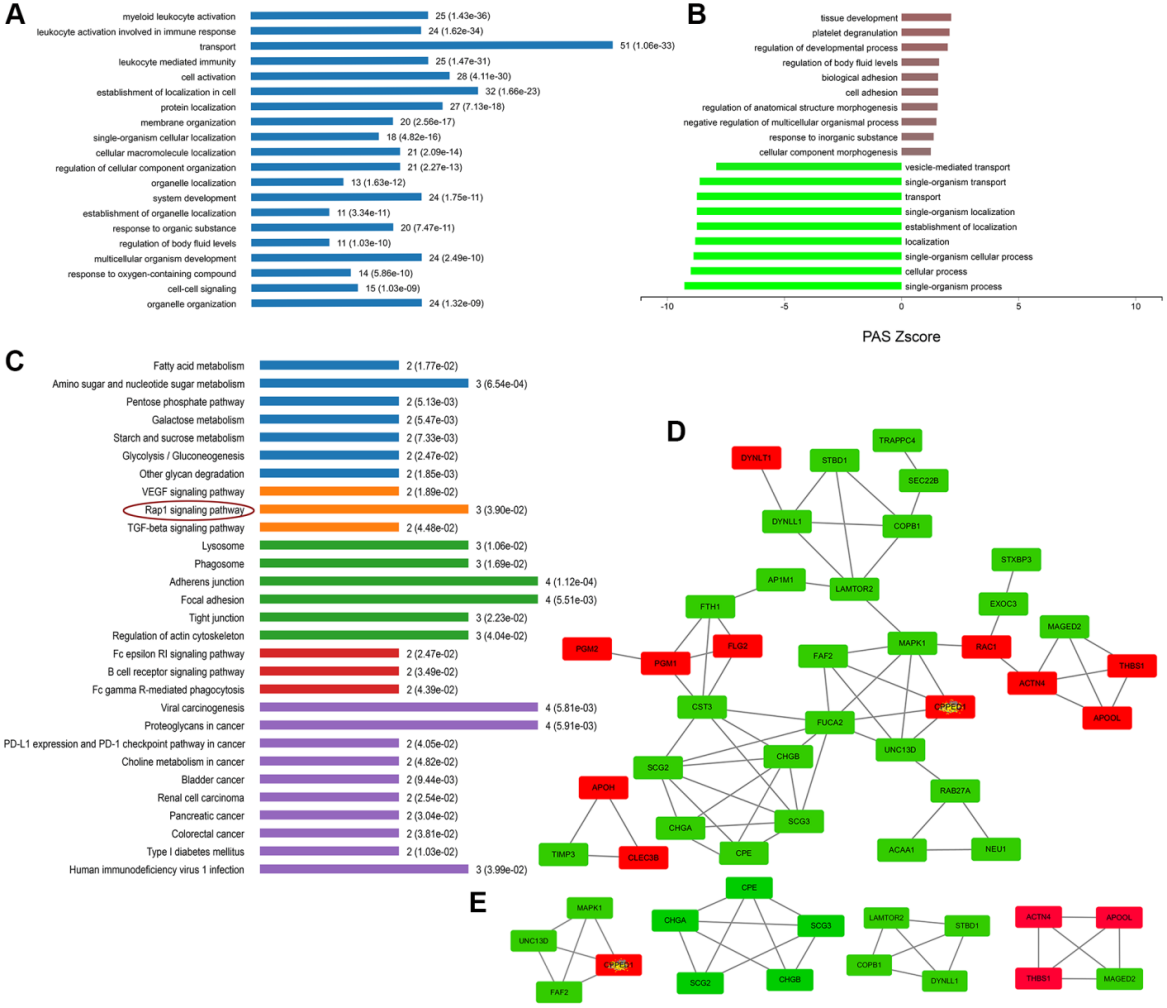
**Enrichment and PPI analysis of 56 significantly differentially expressed proteins on the secretory granules.** (**A**) The involved biological processes (BP) of the 56 secretory granule proteins in Gene Ontology (GO) analysis. (**B**) The dysregulated BP indicated by GO analysis. (**C**) Kyoto Encyclopedia of Genes and Genomes (KEGG) pathways of the 56 secretory granules proteins. (**D**) protein-protein interactions (PPI) network of 56 SGs proteins constructed by the Search Tool for the Retrieval of Interacting Genes (STRING) database. (**E**) Clustering analysis was conducted by molecular complex detection (MCODE) of Cytoscape software.

As expected, the term “secretory granules” was highly enriched in the cell component (CC) of GO analysis. 56 proteins are shown to be located on the SGs referring to the GO term “secretory granules” (GO:0030141). The expressions of these 56 proteins were further verified using real-time PCR. As Figure 5 shown, the mRNA variations of 56 proteins were mostly in consist with their protein levels in the mass spectrometry, except for DBH, MTMR2, and THBS1. DBH and MTMR2 were increased by about 1.5 folds on mRNA levels, while their proteins were decreased. And the mRNA expression of THBS1 remained unchanged, while it was increased by 2.5 folds on the protein level (Figure 5). We suppose the differences might result from the post-transcriptional regulations during their translations. Next, further analysis was conducted on the 56 proteins. According to the GO analysis, we found that most of these affected proteins were mainly enriched in the biological process of transport, followed by the localization in cells, cell activation, and leukocyte mediated immunity (Figure 6A). Furthermore, many biological processes, especially vesicle-mediated transport, were downregulated in SH-sy5Y cells after treatment with MPP^+^ (Figure 6B). To further identify the significant differences between the two groups, a KEGG analysis was performed. Our result showed that corresponding to the result of total significantly differentially expressed proteins, the lysosome, and phagosome functions were largely compromised as well as lipid metabolism. These pathways mainly included AP1M1, NEU1, PPT1, and THBS1, RAC1, MAPK1, and ACAA1, PPT1, respectively (Figure 6C). Next, the protein-protein interactions (PPI) network of 56 SGs proteins was constructed using the Search Tool for the Retrieval of Interacting Genes (STRING, https://string-db.org) Database and further analyzed and visualized with Cytoscape software (https://cytoscape.org) (Figure 6D). After the molecular complex detection (MCODE), four screen modules were found (Figure 6E). Notably, we found that the marker proteins Scg2 and Scg3 were clustered with two members of the chromogranin/secretogranin family CgA (CHGA) and CgB (CHGB) in the second module, all of which are considered to play an essential role in the aggregation and processing of peptide neurotransmitters in the SGs. The above result suggests that several SGs proteins were dysregulated, particularly chromogranins/secretogranins, which might contribute to the lesion of the cells injured by MPP^+^.

### The secretory granules dysfunction may lead to disabled processing and trafficking of secretory proteins in the MPP^+^-induced cell model of Parkinson’s disease

**Figure 7:**
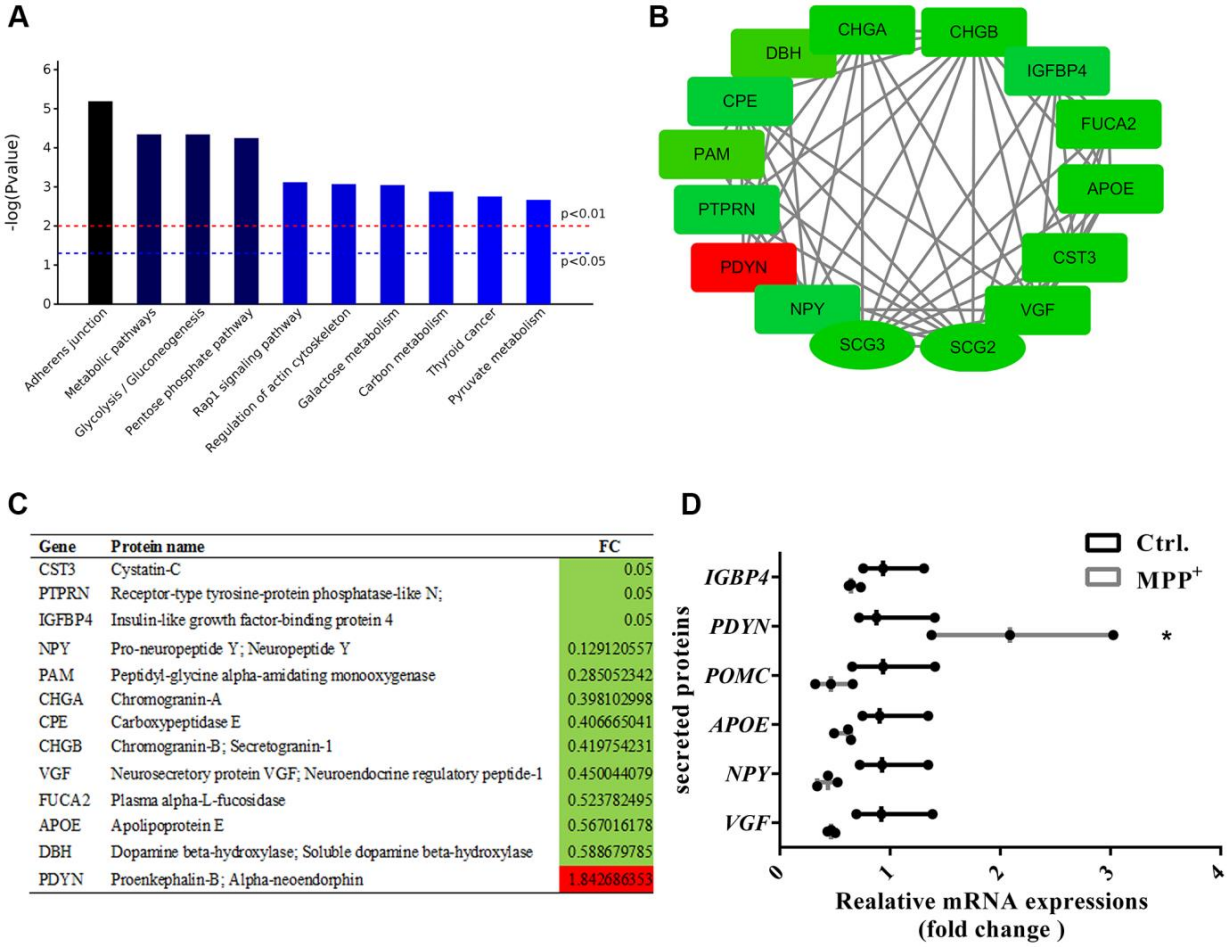
**Enrichment and PPI analysis of 56 significantly differentially expressed secretory granule proteins and 140 secreted proteins.** (**A**) The top 10 enriched KEGG pathways of the 56 secretory granules proteins and 140 secreted proteins. (**B**–**C**) Candidate proteins interacting with secretogranins in peptide processing in SGs indicated by the PPI network. (**D**) The expressions of some secretogranins-related candidate neuropeptides and neuroendocrine hormones are verified using real-time PCR. Two-tailed unpaired Student *t*-tests were performed between the control and treated groups. ^*^Statistically significant with *P* < 0.05; Error bars are SD; *N* = 3.

SGs play crucial roles in the releasing of multiple secreted proteins in the regulated secretory pathway. Next, we sorted out the 140 secreted proteins referring to the CC classification that comprise “extracellular ^**^” and make a combined analysis with the 56 proteins. In the secreted proteins, there were 43 upregulated proteins and 94 down-regulated proteins. KEGG pathway analysis implies that these proteins were mainly clustered in the pathways of the adherent junction, endocytosis, glycolysis and gluconeogenesis, and Rap1 signaling pathway (Figure 7A). After PPI analysis of these proteins, we identified several candidate secreted proteins and peptides that participate in the interaction of the secretogranins-related modules, neurosecretory protein VGF, neuropeptide Y (NPY), Apolipoprotein E (APOE), and prodynorphin (PDYN). And the expressions of the above and some Scg3 related were verified from mRNA levels using real-time PCR (Figure 7B–7D). Interestingly, all these proteins are shown to be down-regulated, except for proenkephalin-B (PDYN), which was two folds increased on both mRNA and protein levels.

## DISCUSSION

An imbalanced availability and sorting disorder of SGs have been identified in a wide array of neurodegenerative disorders. In Alzheimer’s disease (AD), SGs were found to aberrantly accumulated in the senile plaque-surrounding astrocytes and dystrophic neurites in the animal model and patients [27]. Similarly, the docking and secretion of BDNF-containing and ATP-containing SGs were remarkably disrupted in astrocytes of Huntington’s disease (HD) [16]. Yet, the impairment of SGs in PD has been rarely studied. In the current study, we have identified an abundance of Scg2 and Scg3 positive SGs in the dopaminergic neurons in both SNpc and VTA regions of the mouse midbrain. However, these SGs in DA neurons were significantly decreased in the MPTP-induced PD mouse model. Previous studies found that administration of MPTP led to reduced levels of ATP and acute injury of the TH-positive neurons in SNpc and VTA of the mouse. Similarly, SGs impairment was observed in both regions in the current study. However, the decrease of secretogranins seems more remarkable in SNpc than that of VTA. Moreover, we observed that Scg3 in TH-positive neurons of VTA was restored in the recovery period of the MPTP-induced PD model (21 days after the last injection). On the contrary, its decrease in TH-positive neurons of SNpc was irreversible (Data not shown). These results suggest that SGs in SNpc DA neurons are more vulnerable to MPTP, and their impairment may contribute to the motor deficits of PD. Correspondingly, a reduced level of marker proteins Scg3 was observed in both SN and CPU regions, where DA neurons’ cell bodies and terminals are situated. These results were recapitulated in the dopaminergic SH-sy5Y cell model of PD induced by MPP^+^. The findings here in MPTP/MPP^+^-induced PD models accord with our previous work in the dopaminergic cell model induced by another parkinsonian toxin PQ, which showed a significant number decrease and trafficking impairment of Scg3 and CgA positive SGs [21]. Interestingly, the disturbance of Scg3 in both animal and cell models of PD seemed more profound than Scg2. A similar phenomenon has also been found in our previous study, in which the up-regulation of Scg3 was a common feature in multiple parkinsonian toxins (MPP+, dopamine, and MnCl_2_) induced astrocyte activation, whereas the level of Scg2 remained unchanged [28]. Taken together, these findings point to a critical role for SGs impairment in the neuropathology of PD. And we assume that the aberrant Scg3 expression is primarily involved in the process.

SGs act as a critical organelle for the biosynthesis and secretion of multiple peptide hormones, neuroeffectors, and neurotransmitters. The biochemical systems that participate in peptide hormone processing comprise prohormones, protease, and enzymes [29]. According to the PPI and clustering analysis of SGs proteins and the secretory proteins, Scg2 and Scg3 were determined as the biggest module clustered with related classic granins CgA and CgB, both of which were significant deceased on mRNA and protein levels. The members of the chromogranin/secretogranin family are considered key adaptors in the aggregation and processing for peptide aggregation in SGs [18]. Therefore, the current study suggests the function of granin-mediated active peptide production in SGs is largely compromised. Meanwhile, many other proteases and enzymes that involved in peptide and prohormone processing of chromaffin and pancreas SGs decreased after SH-sy5Y cell was exposed to MPP^+^, for instance, carboxypeptidase E (CPE), protein-tyrosine-phosphatase (PTPRN) peptidyl-glycine alpha-amidating monooxygenase (PAM), insulin-like growth factor-binding protein 2 and 4 (IGFBP2 and 4). Correspondingly, we observed the dysregulations of some known peptides that related to SGs besides classical granins, among which the levels of neurosecretory protein (VGF)/NERP-1/NERP-2 and pro-NPY/NPY decreased, while proenkephalin-B/dynorphin increased. VGF is a polypeptide found in SGs of neuronal and neuroendocrine cells. Its precursor and derived peptides are released in response to depolarizing signals through the regulated secretory pathway. Previous studies found that VGF-derived peptide TLQP62 was capable of promoting neurogenesis and synaptic plasticity through brain-derived neurotrophic factor (BDNF) and its receptor tyrosine receptor kinase B (TrkB) [30]. Notably, recent studies found that VGF in the GABA neurons was significantly reduced in PD rat models induced by 6-hydroxydopa as early as three weeks, consistent with the low level of VGF in plasma of PD patients. The declined VGF immunoreactivity showed a linear correlation to the disease duration and olfactory dysfunction and could be restored after levodopa treatment [31]. In addition, decreased levels of VGF-derived peptides TPGH and NERP-1were also identified in the parietal cortex of PD patients in the autopsy [32]. Combined with the above results, we suppose the defective SGs in the processing VGF and its derived peptides might give some novel insights into the pathogenesis of PD. Finally, the expressions of several newly identified neurotransmitters and neurological disease-related proteins also altered significantly, including hippocampal cholinergic neurostimulating peptide (PEBP1), fibroblast growth factor (FGF1), thymopoietin (THBS1), the amyloid-beta (Aβ) A4 precursor protein-binding family B member 1 (APBB1) and apolipoprotein E (APOE) in AD [33–36], Huntingtin protein (HTT) in HD [37], along with the protease inhibitor cystatin C is in epilepsy [38]. Recent proteome studies indicate that FGF2 facilitated the extracellular vesicle (EV)-mediated alpha-synuclein (a-Syn) transmission by upregulated Ras-associated binding proteins (Rabs) and a-Syn interacting proteins in PD and Lewy body pathology (LBP). In the current study, although Rabs were not significantly altered, we observed increased levels of FGF1 and one of the a-Syn interacting proteins ribosomal protein Rsp14, which contributes to mitochondrial fragmentation and energy failure [39, 40]. Brekk et al. [28] identified an aberrant accumulation of α-Syn specific to Scg2 positive SGs in multiple brain regions in the aged mice, accompanied by the loss of synaptic proteins PSD-95 and TUBB3. Therefore, we suppose that the FGF upregulation might play as a shared mechanism to impair neurons’ intra- and extracellular secretory machinery and contribute to the aggregation of α-Syn in PD.

Besides, the proteomics analysis suggested that lipid metabolism and lysosome disorders are two of the main characteristic features of the PD cell model according to the KEGG analysis. Meanwhile, the vesicle-mediated transport and location functions of SGs were largely affected. Previous studies found that lipids facilitate the budding, biogenesis, and exocytosis events of SGs, and participate in intragranular conditions and protein recruitment in the organelle [41]. Like the chromaffin SGs from the bovine adrenal medulla [42], several lipids metabolizing enzymes were identified in the SGs in SH-sy5Y dopaminergic neurons, which showed down-regulated expression levels. We found that several fatty acid metabolism and biosynthesis involved pathways were profoundly downregulated as well, such as biosynthesis of unsaturated fatty acids, fatty acid degradation, and fatty acid metabolism. Notably, phospholipase D3 and cholesteryl ester hydrolase were half decreased in the current study, which plays a vital role in producing and regulating two main lipids necessary in the lipid-mediated mechanisms for SGs phosphatidic acid (PA) and cholesterol [43, 44]. It has been proved that blocking PA synthesis by phospholipase D (PLD) altered the structure of the Golgi apparatus and quantitatively inhibited the secretion of growth hormone [44]. Meanwhile, depletion of cholesterol with lovastatin and methyl-beta-cyclodextrin (mβCD) respectively inhibits the SGs formation and impaired Scg3 and CgA to bind to the membrane of SGs in AtT-20 cells [20]. Considering that anchoring of Scg3 to SGs depends on the cholesterol content of the membrane, we suppose the lipid and cholesterol metabolism disorder here may account for the increased level of Scg3 that drops off into the conditioned medium in Figure 3. The unbalanced cellular lipid content in PD may affect the budding, transport, and protein processing function of SGs. Likewise, several lysosomal related proteins are also found dysregulated in our data, namely palmitoyl protein thioesterase 1 (PPT1), cathepsin F (Cat F), adapter protein complex 1 (AP-1). Previous studies suggest that several missorted proteins and lysosomal hydrolases, including cathepsin B and L, are inadvertently co-packaged into the immature SGs (ISGs) in endocrine cells [45–47]. By budding off of constitutive-like vesicles, the ISGs removed lysosomal enzymes and become mature. And this process is under the recruitment of AP-1 in a casein kinase II-phosphorylation-dependent manner [48, 49]. Hence, we consider that impaired SGs maturation contributes to lysosome dysfunction in PD. Notably, although Cat F inactivation causes a defected lysosomal storage and progressive neurological features in mice, a similar function has yet been reported in Cat F in processing neuropeptides and AD-related neurotoxic Aβ as cathepsin L and cathepsin B [46, 50, 51]. Further studies are needed for its involvement of proteolysis in SGs.

Besides, three of the 56 significantly differentially expressed proteins in “secretory granules” have also been identified or predicted in other omics studies of PD. Among them, ferritin heavy chain (FTH1), which was significantly decreased in our study (0.54, *P* = 0.03), has ever been identified as a differentially expressed gene (DEGs) in the RNA sequencing of PD rat model established by stereotactic 6-hydroxydopamine injection [52]. As the primary regulator of ferritin, FTH1 subunit plays an essential role in iron oxidation and iron balance maintenance. The decrease of FTH1 leads to reduced iron storage and ferroptosis in cells [53]. Moreover, FTH1 was found to be involved in ferritinophagy in 6-OHDA-treated PC-12 cells, whose knockdown results in the inhibited cell viability and mitochondrial dysfunction [52]. The individual profiling on mRNA and protein levels suggests that the interference of FTH1 and the ferroptosis and ferritinophagy pathways might be involved in the PD progression. Calmodulin 3 (CALM3) encodes a family of proteins that bind calcium and function as an enzymatic co-factor. It participates in the regulation of the cell cycle and cytokinesis [54]. The dysregulation and aberration of the CALM3 gene have ever been studied in AD and HD [55, 56]. However, its role in PD remains unclear. In the SGs component analysis of the cell model, we found that CALM3 profoundly increased (1.98, *P* = 0.002). Interestingly, the result is consistent with the RNA microarray expression data of PD-affected SN samples (https://www.ncbi.nlm.nih.gov/geo, GSE54282). Moreover, CALM3 (1.23, *P* < 0.05) was predicted as one of the prioritizing genes for PD after co-analysis of DEGs from the above microarray with the established PD associated-genes (PDAG) using a network science approach [57]. Therefore, the upregulations of CALM3 observed from both cell model and patients tissue make it more convincing to make a further study on its role in PD. Likewise, apolipoprotein H (ApoH) was reported as the biomarker of PD in previous proteomics analyses of the CSF of PD patients [58, 59]. However, it showed the level of apoH in the CSF was largely decrease (0.66, *P* < 0.05) compared to the control group, which was contrary to the increased level of the intracellular apoH in DA neurons indicated by our findings. We suppose that the difference could result from the discrepancy of samples and testing methods. However, considering that ApoH can be secreted, the conflicting results might also imply an intracellular block of trafficking and releasing of ApoH in PD. In short, we believe the integrative analysis based on different omics could help to provide more details of the molecular mechanism and accelerate the identification of biomarkers for PD.

## CONCLUSIONS

Recent years have seen studies linking PD pathogenesis to vesicle transport dysfunctions. The present study connects the PD to the disorder of SGs, a class of large vesicles for the biosynthesis and regulated secretion of neuropeptides. We observed loss of Scg2 and Scg3-positive SGs in DA neurons of MPTP mouse models. Meanwhile, the level of Scg3 was remarkably decreased in CPU and SN regions and MPP^+^-treated SH-sy5y cells, which suggests its involvement in the SGs impairment. Furthermore, the proteomic study of SGs has provided the protein architecture of the impaired regulated secretory vesicle system. The biochemical analysis has indicated the disabled lysosome and peroxisome, lipid, and energy metabolism disorders are three characteristic features of the PD cell model, which may disturb vesicle-mediated transport and location functions of SGs. In addition, the peptide and neurotransmitter processing function of SGs mediated by chromogranin/secretogranin is remarkably compromised in PD, which results in the dysregulations of several related proteins and their active peptides, namely VGF, NPY, APOE, and proenkephalin-B. Combined with the previous omics study, we suggest that three SGs proteins screened in the current study, FTH1, CALM3, and ApoH are promising to be the biomarker of PD. A limitation of the present study is the absence of wet-lab experimental data, especially for validating Scg2 and Scg3 expressions in PD samples. Further studies are needed to elucidate the active processing of these candidate proteins in SGs of DA neurons and their involvement in PD, particularly the proteolysis of VGF peptides and the role of Scg3 during the process. Taken together, the present proteomic study has provided an extensive proteinogram of SGs. It is helpful to understand the molecular mechanisms in the disease better.

## Supplementary Materials

Supplementary Figures

Supplementary Table 1
