# Supplementary material for: Proteomic characterization of secretory granules in dopaminergic neurons indicates chromogranin/secretogranin-mediated protein processing impairment in Parkinson’s disease
Source: Aging (Albany NY). 2021 Aug 21;13(16):20335–58. doi: 10.18632/aging.203415 (PMC8436928; doi:10.18632/aging.203415)
Supplement: Supplementary Figures [file aging-13-203415-s001.pdf]

SUPPLEMENTARY FIGURES

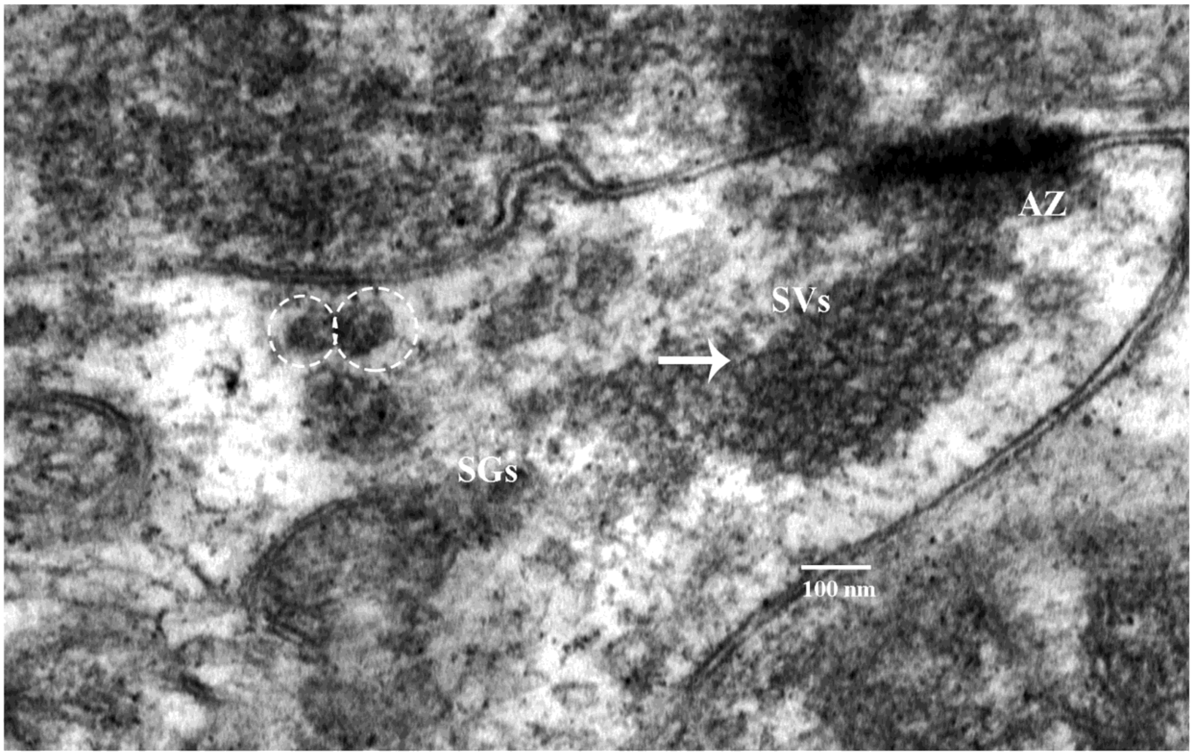

Supplementary Figure 1. Electron microscopy image of dense-core SGs of neurons in SN. The existence of SGs was captured using electron microscopy. Several dense-core SGs are present at the border of synaptic vesicle clusters. SVs: Synaptic vesicles; AZ: Active zone; SGs: Secretory granules.

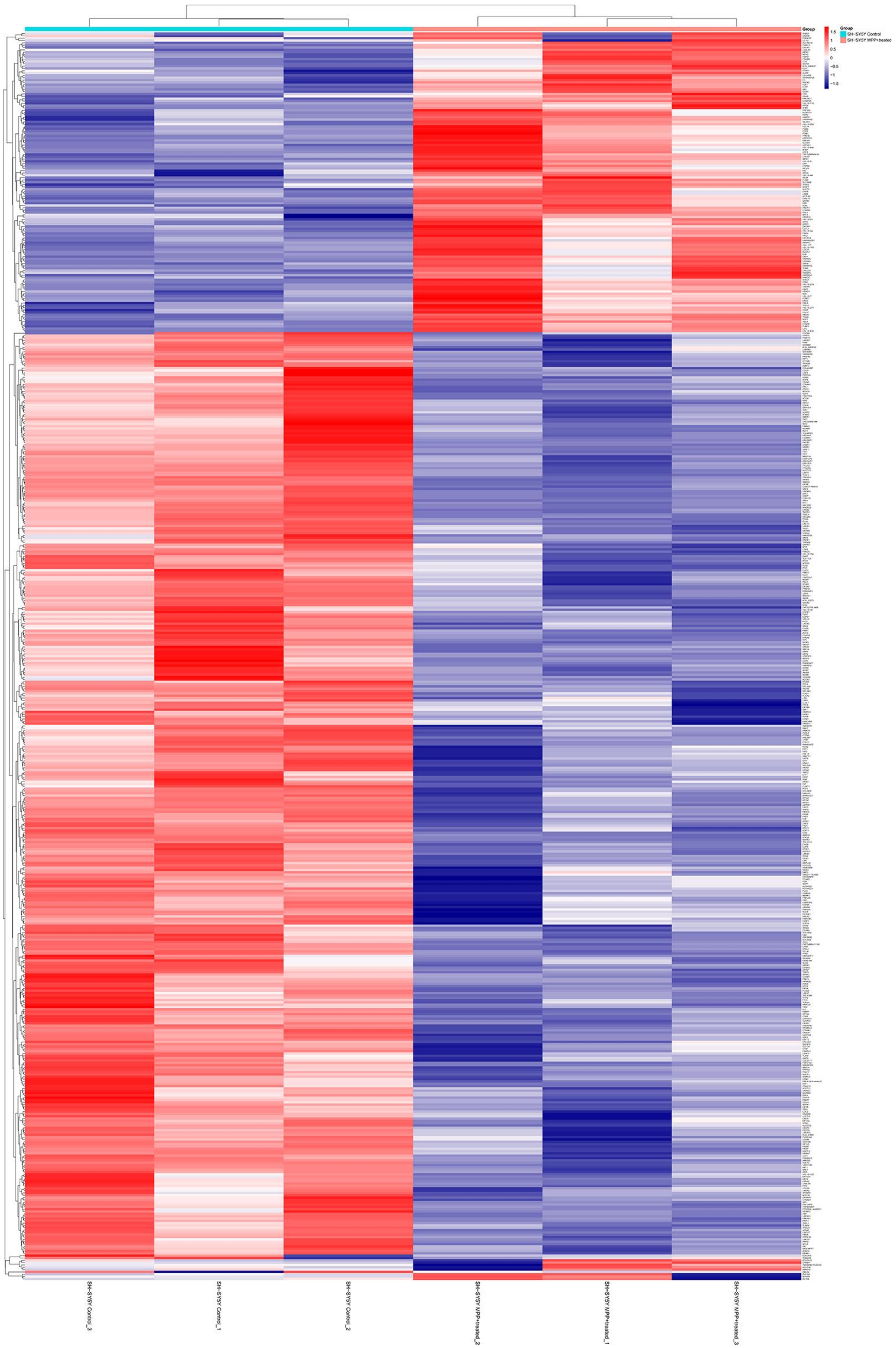

Supplementary Figure 2. Heatmap analysis using complete linkage hierarchical clustering of the significantly differentially expressed proteins between the control and MPP^+ groups. Cluster 1 represents significantly up-regulated proteins in and MPP^+ treated groups, cluster 2 represents significantly down-regulated proteins. *N* = 3.

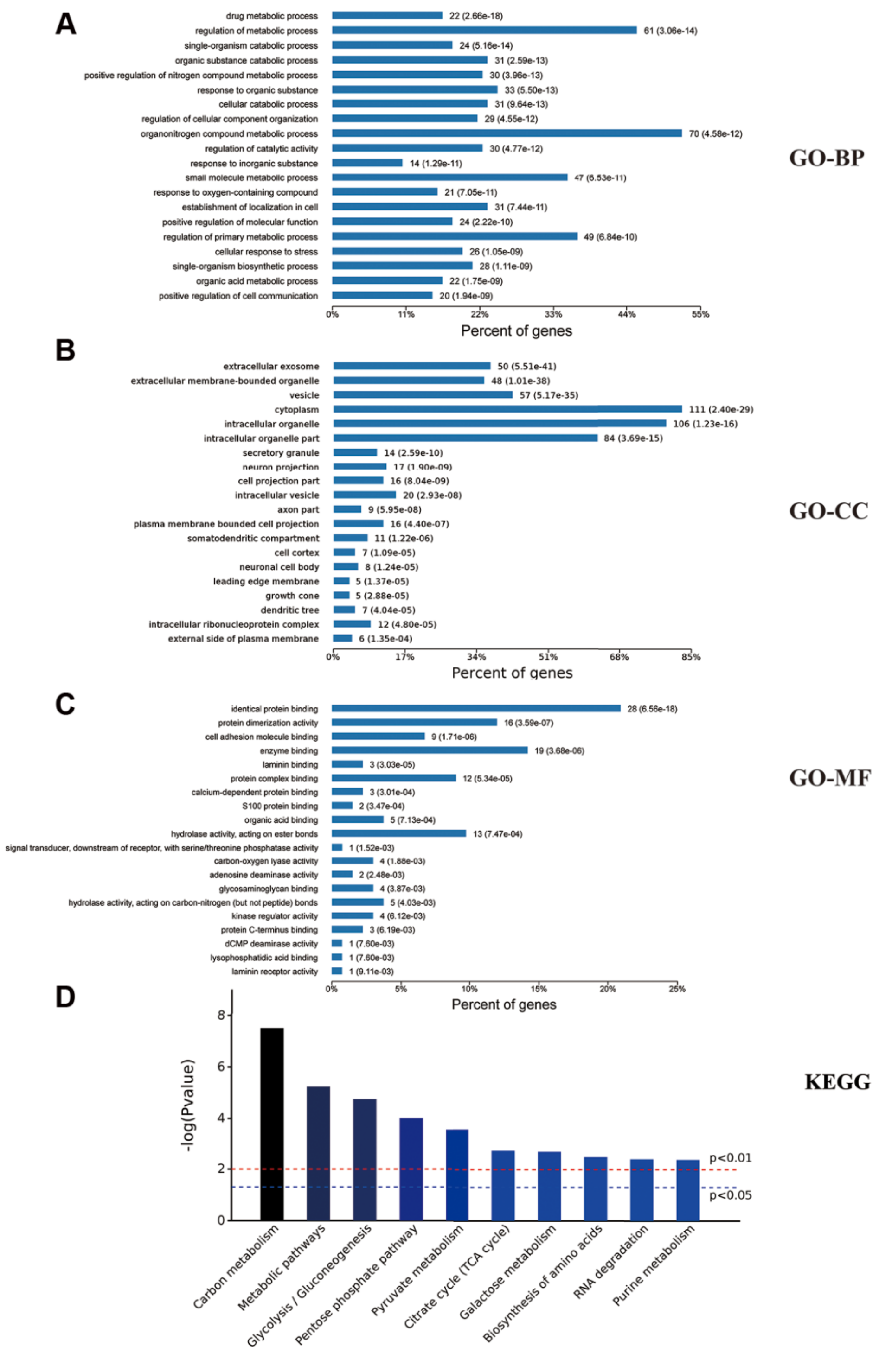

Supplementary Figure 3. Enrichment analysis of significantly up-regulated expressed proteins in the secretory granules enriched samples of control and MPP$^+$ groups. The secretory granules enriched samples of control and MPP$^+$ treated SH-sy5Y cells were separated via sucrose density gradient centrifugation, and sent for the mass spectrometry analysis. 145 proteins were identified as significantly increased with the fold of change (FC ≥ 1.5, $P < 0.05$, $N = 3$). And Gene ontology and Kyoto Encyclopedia of genes and genomes pathways analysis was conducted to these proteins. (**A**) Gene ontology biological processes (BP). (**B**) Gene ontology cellular components (CC). (**C**) Gene ontology molecular functions (MF). (**D**) Kyoto Encyclopedia of genes and genomes pathways.

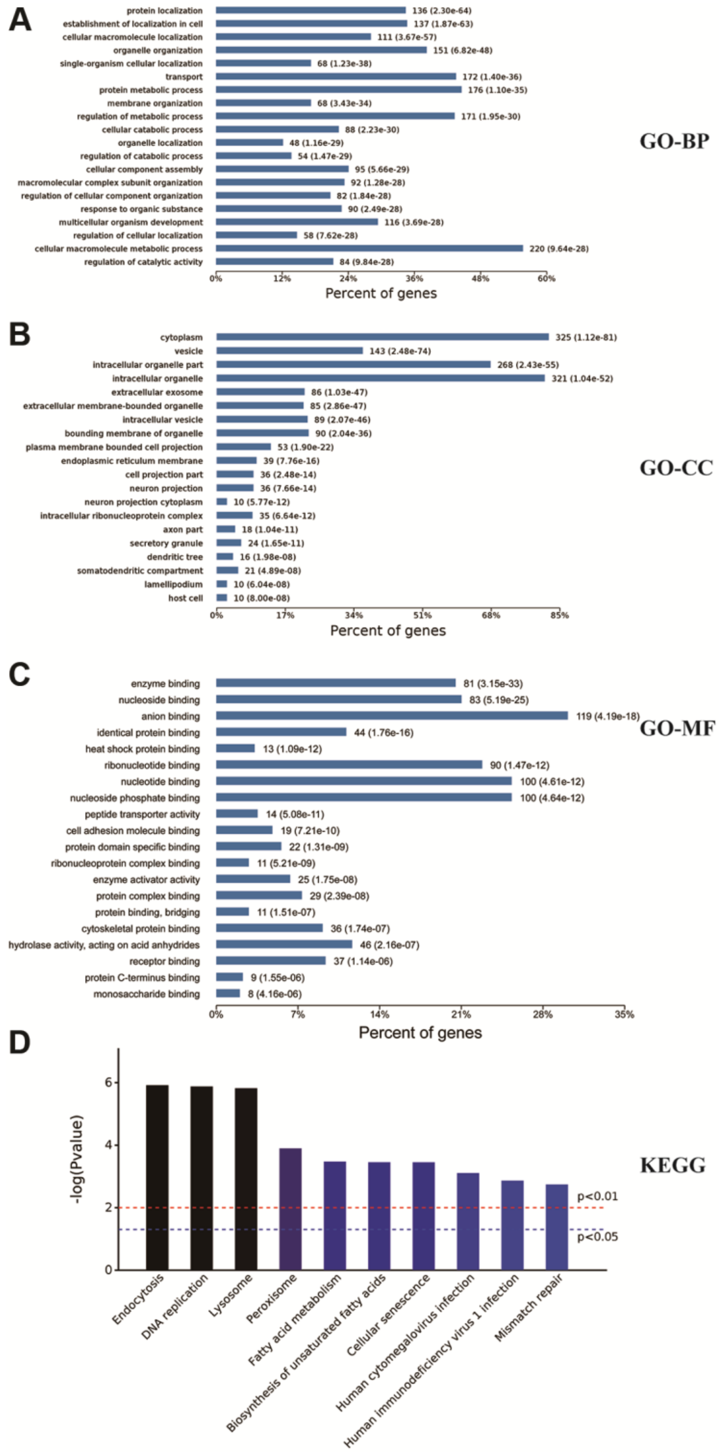

Supplementary Figure 4. Enrichment analysis of significantly down-regulated expressed proteins in the secretory granules enriched samples of control and MPP+ groups. The secretory granules enriched samples of control and MPP+ treated SH-sy5Y cells were separated via sucrose density gradient centrifugation, and sent for the mass spectrometry analysis. 576 proteins were identified as significantly decreased with the fold of change (FC ≥ 1.5, $P < 0.05$). And Gene ontology and Kyoto Encyclopedia of genes and genomes pathways analysis was conducted to these proteins. (**A**) Gene ontology biological processes (BP). (**B**) Gene ontology cellular components (CC). (**C**) Gene ontology molecular functions (MF). (**D**) Kyoto Encyclopedia of genes and genomes pathways.
